# Bendamustine plus rituximab in Japanese patients with relapsed or refractory diffuse large B-cell lymphoma

**DOI:** 10.1007/s00277-022-04801-2

**Published:** 2022-03-04

**Authors:** Kayoko Murayama, Toru Kiguchi, Koji Izutsu, Yoshihiro Kameoka, Michihiro Hidaka, Harumi Kato, Shinya Rai, Junya Kuroda, Kenichi Ishizawa, Satoshi Ichikawa, Kiyoshi Ando, Michinori Ogura, Koji Fukushima, Yasuhito Terui

**Affiliations:** 1 Department of Hematology, Gunma Prefectural Cancer Center, Ota, Japan; 2 Department of Hematology, Chugoku Central Hospital, Fukuyama, Japan; 3 Department of Diabetes, Endocrinology and Hematology, Dokkyo Medical University Saitama Medical Center, 2-1-50, Minami-Koshigaya, Koshigaya, Saitama 343-8555 Japan; 4 Department of Hematology Tokyo, National Cancer Center Hospital, Tokyo, Japan; 5 Department of Hematology, Nephrology and Rheumatology, Akita University Graduate School of Medicine and Faculty of Medicine, Akita, Japan; 6 Department of Hematology, National Hospital Organization Kumamoto Medical Center, Kumamoto, Japan; 7 Department of Hematology and Cell Therapy, Aichi Cancer Center Hospital, Nagoya, Japan; 8 Department of Hematology and Rheumatology, Kindai University Faculty of Medicine, Osakasayama, Japan; 9 Division of Hematology and Oncology, Department of Medicine, Kyoto Prefectural University of Medicine, Kyoto, Japan; 10 Department of Internal Medicine III, Yamagata University Faculty of Medicine, Yamagata, Japan; 11 Department of Hematology and Rheumatology, Tohoku University Graduate School of Medicine, Sendai, Japan; 12 Department of Hematology & Oncology, Tokai University School of Medicine, Isehara, Japan; 13 Department of Hematology and Oncology, Kasugai Municipal Hospital, Kasugai, Japan; 14 Medical Affairs Department, SymBio Pharmaceutical Ltd., Research & Development Division, Tokyo, Japan; 15 Department of Hematology Oncology, Cancer Institute Hospital of JFCR, Tokyo, Japan; 16 Department of Hematology, Saitama Medical University, Saitama, Japan

**Keywords:** Bendamustine hydrochloride, Lymphoma, Large B cell, Diffuse, Recurrence, Rituximab, Salvage therapy

## Abstract

This single-arm phase 3 study was conducted to confirm the results of our phase 2 study of bendamustine (B)-rituximab (R) in patients with relapsed/refractory diffuse large B cell lymphoma (rrDLBCL). The primary endpoint was overall response rate (ORR). Autologous stem cell transplantation-ineligible rrDLBCL patients with ≤ 2 prior chemotherapy regimens received R 375 mg/m^2^ IV on day 1 and B 120 mg/m^2^/day IV on days 2 and 3 every 21 days up to 6 cycles. Thirty-eight patients with a median age of 74 years (range, 43–86) received BR. The ORR and complete response rates were 76.3% and 47.4%, respectively. With a median follow-up of 19.5 months including long-term follow-up, median progression-free survival was 11.9 months. Median OS was 29.2 months. Discontinuation of treatment due to Gr3-5 TEAE was observed among 13 of 38 patients (34.2%). One patient with cytomegalovirus enterocolitis died during follow-up. This BR regimen was confirmed to be effective and tolerable in studied patients. ClinicalTrials.gov Identifier: NCT03372837 registered on 14 December 2017, NCT04354402 registered on 21 April, 2020.

**Supplementary Information:**

The online version contains supplementary material available at 10.1007/s00277-022-04801-2.

## Introduction

Diffuse large B cell lymphoma (DLBCL) is the most common subtype of non-Hodgkin lymphoma (NHL) in Western countries and Japan [1]. Rituximab (R) in combination with cyclophosphamide, doxorubicin, vincristine, and prednisolone (R-CHOP) is the current standard first-line treatment for patients with DLBCL [2], with a cure rate of 50–60% [3]. Despite improvements in the prognosis of patients with DLBCL in the R era, however, relapsed or refractory DLBCL (rrDLBCL) developing in approximately one-third of patients with DLBCL remains a major cause of morbidity and mortality [4]. Patients with rrDLBCL eligible for high-dose chemotherapy (HDC)/autologous stem cell transplantation (ASCT) are usually treated with intensive salvage regimens including R, dexamethasone, high-dose cytarabine, and cisplatin (R-DHAP); R, ifosfamide, carboplatin, and etoposide (R-ICE); R, gemcitabine, dexamethasone, and cisplatin (R-GDP); or R, cyclophosphamide, high-dose cytarabine, etoposide, and dexamethasone (CHASER) [5]. Patients who respond to these salvage regimens undergo ASCT. In patients ineligible for ASCT who are mainly composed of the elderly, by contrary, these salvage regimens are usually associated with severe toxicities. In recent years, a number of novel immunotherapeutic modalities for rrDLBCL (e.g., immune checkpoint inhibitors, chimeric antigen receptor T-cell [CAR-T] therapy, and antibody–drug conjugates [ADCs]) have been under investigation, some of which have been approved for the treatment of rrDLBCL when used alone or in combination with combined chemotherapies. For example, CAR-T therapy has shown efficacy for rrDLBCL, and 5 drugs including axicabtagene have received regulatory approval from the Food and Drug Administration. However, those novel therapies still present challenges with respect to accessibility, patient’s functional status, disease burden, and price. Thus, an optimal salvage regimen for them remains as an unmet need.

Previously, we reported the results of a phase 2 study of the bendamustine-R (BR) regimen for ASCT-ineligible patients with rrDLBCL in Japan and Korea [6]. The study showed an overall response rate (ORR) of 62.7% and a complete remission (CR) rate of 37.3%. Based on its results, the BR regimen is now listed as one of treatment options for rrDLBCL in international guidelines (e.g., National Comprehensive Cancer Network guidelines on B cell lymphomas [7]) and is also used as a common backbone for the combination with novel agents including polatuzumab vedotin, although a different dose of B is used [8]. Nevertheless, the regulatory approval of the BR regimen for rrDLBCL was not obtained because the regulatory authority in Japan deemed the phase 2 study exploratory. Therefore, we conducted this single-arm phase 3 study to confirm the results of the phase 2 study in an attempt to meet a requirement of the regulatory authority.

## Material and methods

### Eligibility criteria, exclusion criteria, and study overview

Patients aged 20 years or older were eligible when they met all of the following inclusion criteria: histopathologically confirmed DLBCL based on the 2008 WHO Classification of Tumours of Haematopoietic and Lymphoid Tissues, 4th edition [9]—in principle, the most recent histopathological specimen was used for diagnosis of the disease in individual patients; positivity of tumor cells to CD20 demonstrated by immunohistostaining or flow cytometry; disease progression, relapse, or recurrence after standard R-CHOP (or R-CHOP-like) as the first-line therapy; measurable lesions (computed tomography [CT]-measured major diameter: > 1.5 cm); a survival expectancy of at least 3 months; Eastern Cooperative Cancer Oncology Group (ECOG) performance status score of 0 or 1; adequate major organ functions defined as those in our previous phase 2 study [6]; and written informed consent to participate in the present study. The key exclusion criteria were transformed lymphoma, refractoriness to any of prior treatments, ≥ 3 in the number of prior regimens, central nervous system infiltration or clinical symptoms suggestive of the infiltration, and serious complications.

Between January 15, 2018, and August 30, 2019, the present study was conducted at 33 medical institutions in Japan after approval of the protocol by the institutional review board (IRB) at each institution and according to the provisions of the Declaration of Helsinki. All patients provided IRB-approved written informed consent prior to the conduct of any study-specific procedures or assessments. The survey ended on November 11, 2020.

### Study design and procedures

Patients received R 375 mg/m^2^ IV on day 1 and B 120 mg/m^2^/day IV on days 2 and 3 of each cycle every 21 days for up to 6 cycles. Patients proceeded to the next cycle when meeting all of the following criteria by day 22 after initiation of the prior cycle; otherwise, next cycle dosing must be deferred: neutrophil count ≥ 1,000/mm^3^, platelet count ≥ 75,000/mm^3^, ALT/AST ≤ 5 times of upper normal limit, total bilirubin < 2.0 mg/dl, serum creatinine < 2.0 mg/dl, and no sustaining grade 3 adverse events. The treatment must be discontinued if the criteria are not met by day 36. In the second and subsequent cycles, the dose of B was reduced when treatment-emergent adverse events (TEAEs; defined as all unfavorable medical events including laboratory abnormalities) that had developed in the previous cycle fell under any of the dose reduction criteria (e.g., grade 4 hematologic toxicity [excluding lymphocyte count decreased]). The long-term follow-up survey was conducted to update the overall survival (OS) of patients who received at least one dose of BR. The use of granulocyte-colony-stimulating factor (G-CSF) was at the discretion of the investigator. Prophylaxis with trimethoprim-sulfamethoxazole and antiviral prophylaxis (e.g., acyclovir) were recommended when CD4 lymphocyte count was ≤ 200/mm^3^. Furthermore, the development of any opportunistic infections (e.g., cytomegalovirus [CMV] infection) was monitored carefully to initiate preemptive treatment with antivirals (e.g., ganciclovir). Prophylaxis for emesis with steroids (e.g., dexamethasone) was not predefined in the present study.

### Study endpoints, assessments, and criteria

The primary endpoint was the ORR including CR and partial remission (PR). The responses assessed were the best responses that patients showed during the treatment. The secondary endpoints included the CR rate, OS (defined as the time from entry onto the study until death of any cause), progression-free survival (PFS; defined as the time from entry onto a study until disease progression or death of any cause), and duration of response (DOR). Response was assessed by CT and [^18^F] fluorodeoxyglucose positron emission tomography according to the revised response criteria for malignant lymphoma [10].

Concerning safety, patients in the safety population were analyzed for TEAEs that were expressed according to Medical Dictionary for Regulatory Activities-Japanese (MedDRA-J) version 22.1, with grading defined according to the National Cancer Institute Common Terminology Criteria for Adverse Events version 4.0 Japan-Clinical Oncology Group.

### Statistical analyses

The threshold and expected ORRs were set to be 35% and 62%, respectively, based on a phase 2 study of R monotherapy in patients with aggressive B cell NHL (ORR: 35%) [11] and our phase 2 study (ORR: 62.7%) [6]. The number of patients, which was required to afford 90% power for confirming that the ORR surpassed its predefined threshold value, was calculated to be 36. The response rate will be 52.8% (95% confidence interval [CI]: 35.5–69.6) when 19 of 36 patients respond to BR, thus allowing to confirm that the lower limit for 95% CI surpasses the threshold value.

The Kaplan–Meier estimates were used to summarize the OS, PFS, and DOR. Regarding inter-stratum differences in the ORR by background factor, Fisher’s exact test was conducted to calculate *p*-values. The efficacy analysis included patients in the efficacy population, and the safety analysis included all patients who received at least one dose of any study treatment. Two-tailed *p*-values were calculated. All statistical analyses were conducted with the SAS® software version 9.4 (SAS Institute, Inc.; Cary, NC).

## Results

### Patients

**Fig. 1:**
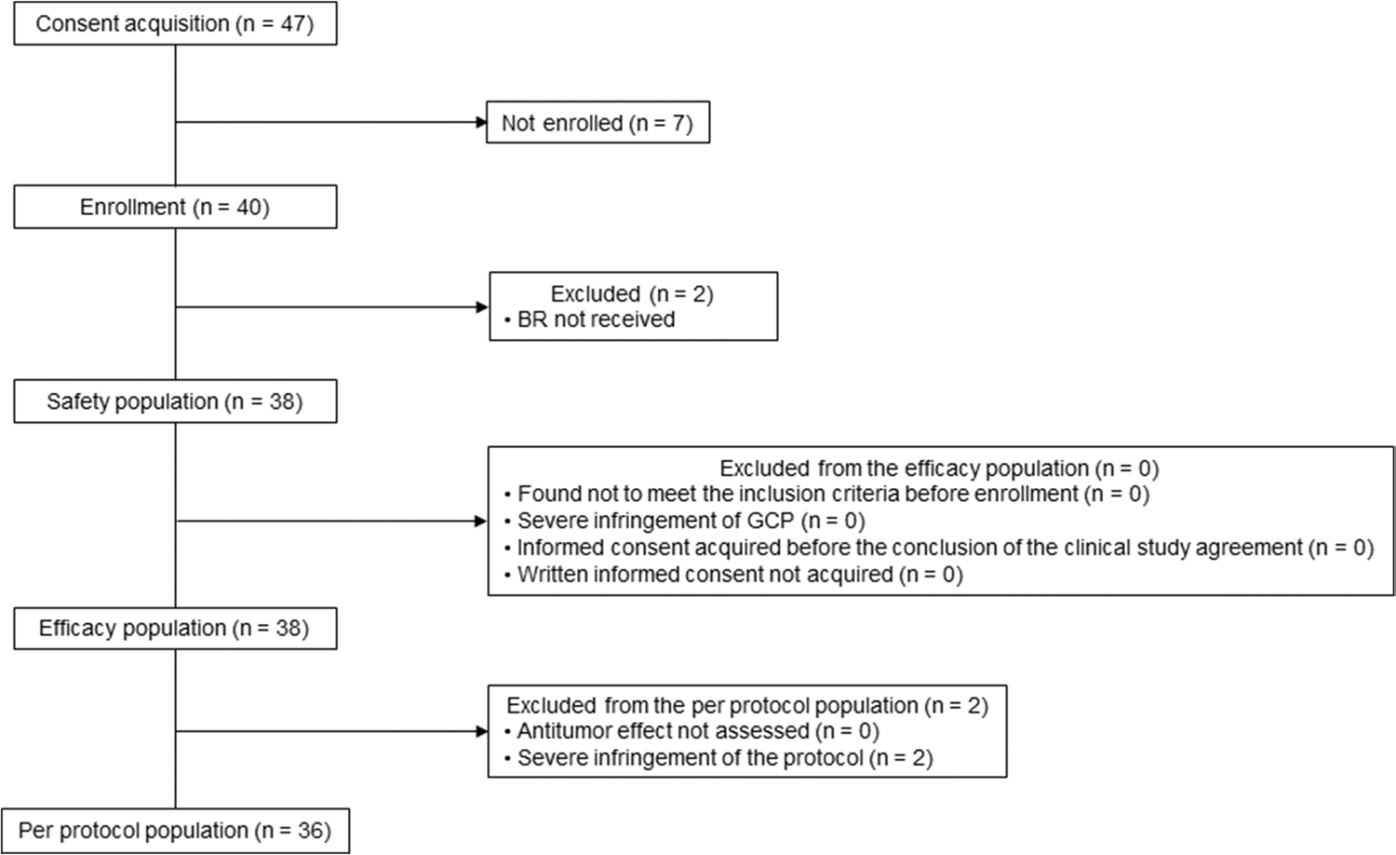
Patient disposition. B, bendamustine; R, rituximab; GCP, good clinical practice

Figure 1 indicates patient disposition. Out of 47 patients who had given written informed consent, 7 of 47 patients were excluded due to the following reasons: no measurable lesion (*n* = 1), no diagnosis of DLBCL (*n* = 3), neutropenia (< 1500/mm^3^) and thrombocytopenia (< 100,000/mm^3^) (*n* = 1), malignant pleural effusion (*n* = 1), investigators discretion (choosing other salvage regimen) (*n* = 1). Two patients did not meet the dosing initiation criteria (neutropenia; *n* = 1, increase of serum creatinine; *n* = 1). Thirty-eight of 47 patients were assessed for efficacy and safety. The median age of 38 patients was 74 years (range: 43–86) (Table 1), and all patients had relapsed DLBCL. Patients with 1 prior chemotherapy regimen were predominant. Two patients were excluded from the per protocol population (excess supportive corticosteroid dosing beyond the predefined criteria and misdiagnosis of the primary disease). The median follow-ups of the present study that ended on August 30, 2019, and of the long-term follow-up survey for OS that ended on November 11, 2020, were 7.8 months (range: 1.3–16.7) and 19.5 months (range: 1.3–31.7), respectively.

**Table 1:** Patient baseline characteristics

	*N* = 38
Age, years, *n* (%)	
Overall, years, median (range)	74 (43–86)
> 75	11 (28.9)
65–75	20 (52.6)
< 65	7 (18.4)
Sex, *n* (%)	
Male	13 (34.2)
Female	25 (65.8)
ECOG performance status, *n* (%)	
0	14 (36.8)
1	24 (63.2)
Number of prior chemotherapy regimens, *n* (%)	
1	23 (60.5)
2	15 (39.5)
Time-to-enrollment since the initiation of first-line therapy, n* (%)	
< 12 months	7 (18.4)
≥ 12 months	16 (42.1)
Prior HDC/ASCT, *n* (%)	
Present	6 (15.8)
Absent	32 (84.2)
Ann Arbor clinical stage, *n* (%)	
I–II	13 (34.2)
III	6 (15.8)
IV	19 (50.0)
Tumor size	
≥ 5 cm, *n* (%)	10 (26.3)
Cell-of-origin category by gene expression profiling, *n* (%)	
• GCB	6 (15.8)
• ABC	9 (23.7)
• Unclassified	5 (13.2)
Hans algorithm, *n* (%)	
• GCB	12 (31.6)
• Non-GCB	23 (60.5)
International prognostic index risk category, *n* (%)	
Low + low intermediate	29 (76.3)
High intermediate + high	9 (23.7)

^*^Includes eligible patients who received 1 regimen; other patients who received 2 regimens or ASCT were not included

*N* total number of patients, *n* number of patients, *ECOG* Eastern Cooperative Oncology Group, *HDC/ASCT* high-dose chemotherapy/autologous stem cell transplantation, *GCB* germinal center B-cell-like, *ABC* activated B-cell-like

### Exposure

The median number of delivered cycles was 4 (range: 1–6). The data on exposure to BR are summarized in Supplementary Table S1. When proceeding to the next cycle, treatment delay occurred due to various reasons (e.g., neutrophil count decreased, < 1,000/mm^3^) in the following patients: 7, 14, 1, 7, and 4 patients in cycles 1, 2, 3, 4, and 5, respectively. Dose reduction occurred due to grade 4 hematologic toxicity (excluding lymphocyte count decreased) (*n* = 13), renal function impairment (*n* = 2), fever, urticaria, acute upper respiratory inflammation, gastric ileus, dehydration, or increase of lipase (*n* = 1 for each) in the following patients: 4, 8, 6, and 3 patients in cycles 1, 2, 4, and 5, respectively. BR was discontinued in a total of 27 patients (71.1%) due to the following reasons: dose modification rules in 9 patients; TEAE irrelevant to dose modification rule in 8 patients (grade 3–5 TEAE in total including hematological toxicity in 13 patients; neutropenia (*n* = 7), thrombocytopenia (*n* = 2, neutropenia and thrombocytopenia *n* = 1), leukocytopenia (*n* = 1; identical patient with neutropenia), liver injury, pneumonia, CMV enteritis, stress cardiomyopathy, pelvic infection (*n* = 1 for each); disease progression or symptom deterioration in 7 patients; more than 2 dose reductions in 1 patient and others in 2 patients.

### Efficacy

**Fig. 2:**
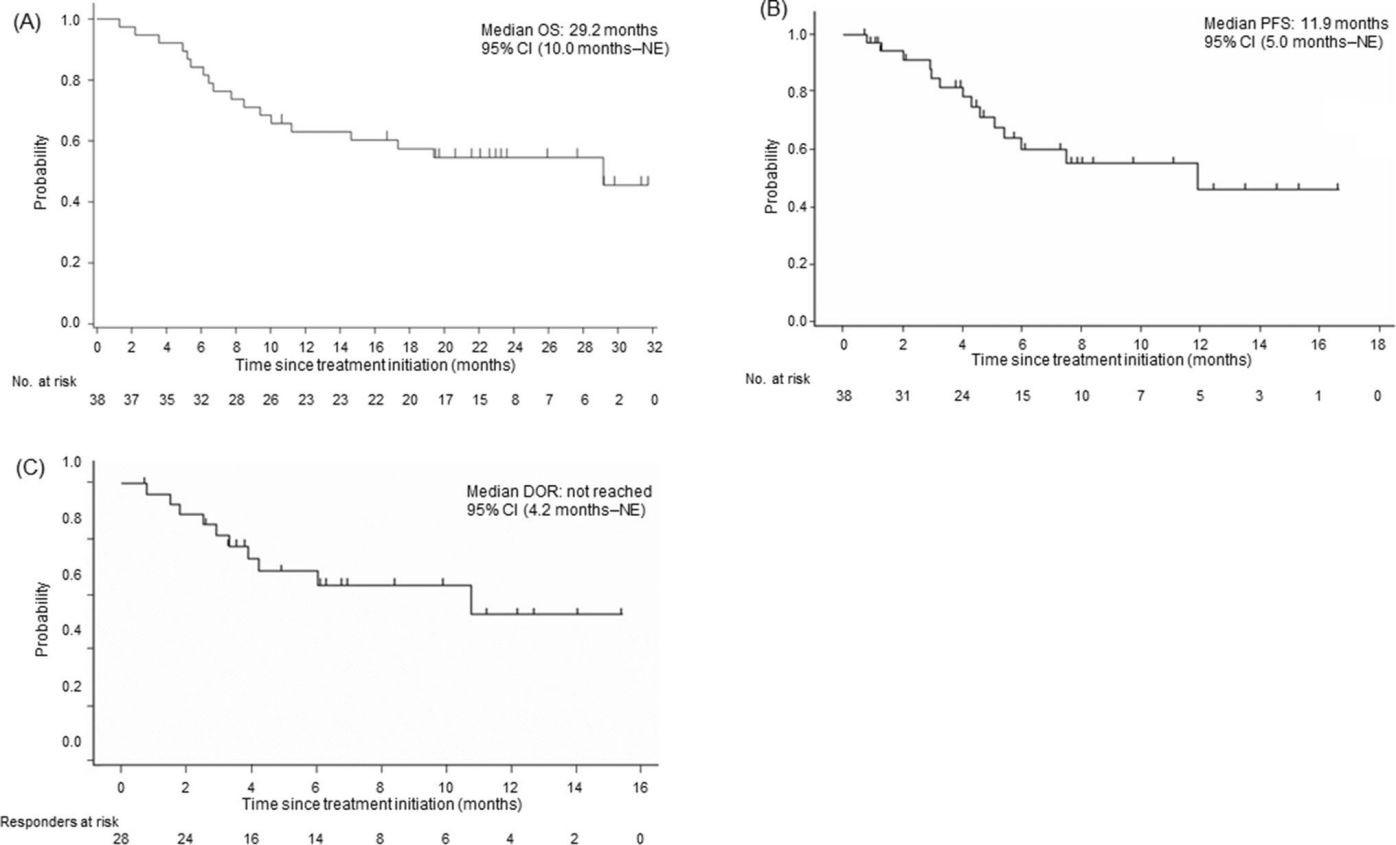
Survival data: as of November 11, 2020—**A** OS; as of August 30, 2019—**B** PFS and **C** DOR. OS, overall survival; NE, not evaluable; PFS, progression-free survival; DOR, duration of response

**Fig. 3:**
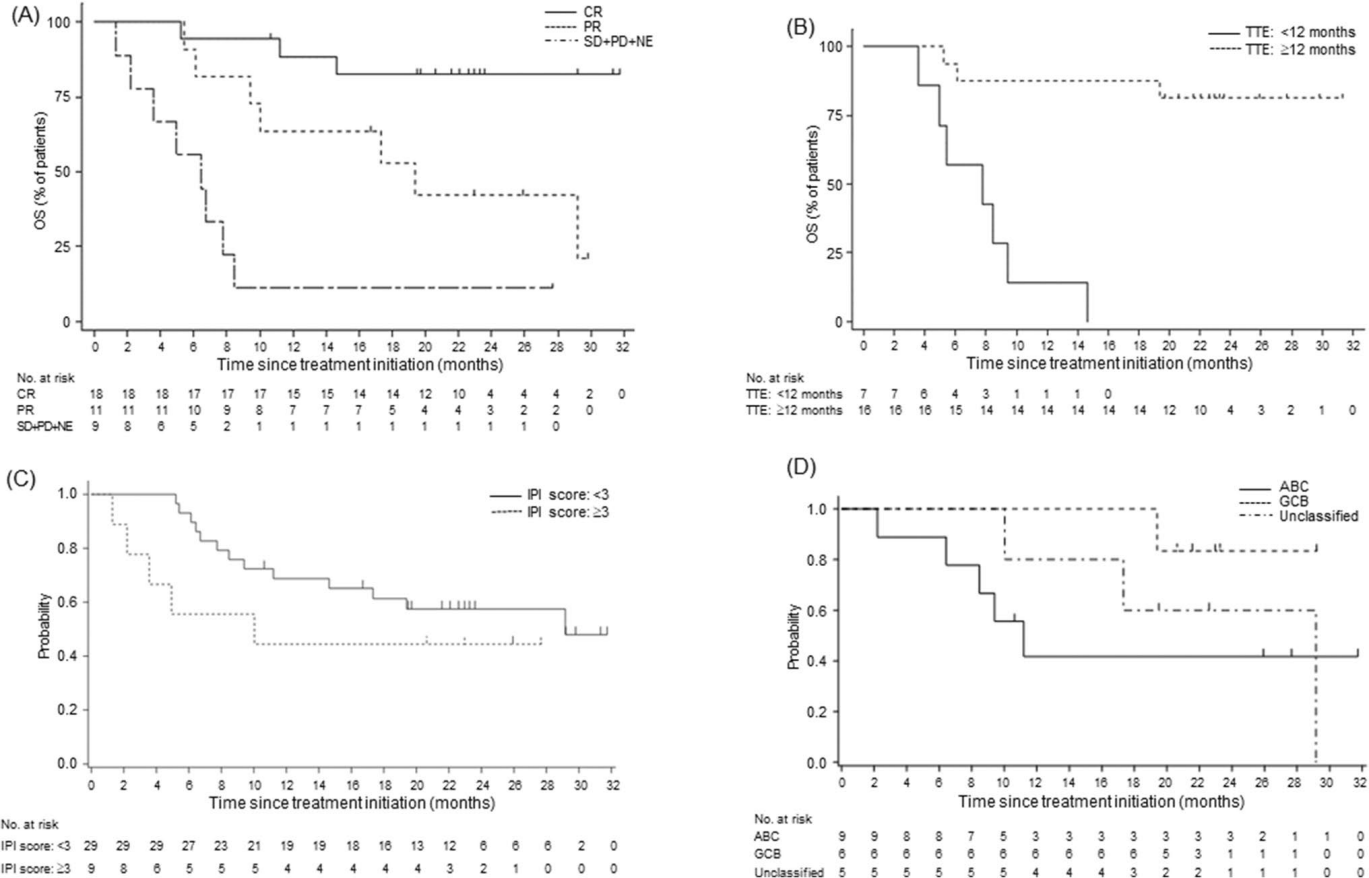
Kaplan–Meier plots of overall survival for the following prognostic factors in the long-term follow-up survey. **A** Response criteria. **B** Time-to-enrollment after the initiation of first-line therapy, which includes eligible patients who received 1 regimen; other patients who received 2 regimens or ASCT were not included. **C** International prognostic index score. **D** Cell of origin. OS, overall survival; CR, complete remission; PR, partial remission; SD, stable disease; PD, progressive disease; NE, not evaluable; TTE, time-to-enrollment; IPI, international prognostic index; ABC, activated B-cell-like; GCB, germinal cell B-cell-like; ASCT, autologous stem cell transplantation

The ORR of 76.3% (95% CI: 59.8–88.6; Table 2) met the primary endpoint. The CR rate was 47.4% (95% CI: 31.0–64.2; Table 2). The total size of target lesions was reduced in 92.1% (35/38) of patients at the time of best overall response assessment. Concerning the secondary endpoints for efficacy, median OS was 29.2 months (95% CI, 10.0 months, not evaluable [NE]; Fig. 2A); median PFS and median DOR were 11.9 months (95% CI, 5.0 months, NE; Fig. 2B) and not reached (95% CI, 4.2 months, NE; Fig. 2C), respectively. The subgroup analysis (Table 2) revealed the higher ORRs in patients with longer time-to-enrollment after the initiation of first-line therapy (< 12 months [42.9%] versus ≥ 12 months [93.8%], *P* = 0.017), lower serum lactate dehydrogenase (< 240 IU/L [88.0%] versus ≥ 240 IU [53.8%], *P* = 0.040), and a lower International Prognostic Index score (< 3 [86.2%] versus ≥ 3 [44.4%], *P* = 0.020). The analysis on the OS of rrDLBCL patients based on response and prognostic factors in the long-term follow-up survey (Fig. 3A–D) revealed a tendency for differences in survival thereof. Five of 6 patients with a previous history of HDC/ASCT responded to BR—3 of whom achieved CR. Among 38 patients, 29 (76.3%) responded to BR by cycle 2, and 17 (44.7%) achieved CR by cycle 4 (Supplementary Figure S1).

**Table 2:** Efficacy

Variables	*n*	Response, *n*					ORR, % [95% CI]	CR rate, % [95% CI]	*P*-value^a^
		CR	PR	SD	PD	NE			
Overall	38	18	11	5	2	2	76.3 [59.8–88.6]	47.4 [31.0–64.2]	
Sex									
Male	13	3	5	3	1	1	61.5 [31.6–86.1]	23.1 [5.0–53.8]	*P* = 0.226
Female	25	15	6	2	1	1	84.0 [63.9–95.5]	60.0 [38.7–78.9]	
Age, years									*P* = 1.000
> 75	11	4	4	2	1	0	72.7	36.4	
							[39.0–94.0]	[10.9–69.2]	
65–75	20	9	6	2	1	2	75.0	45.0	
							[50.9–91.3]	[23.1–68.5]	
< 65	7	5	1	1	0	0	85.7	71.4	
							[42.1–99.6]	[29.0–96.3]	
Number of prior chemotherapy regimens									
1	23	11	7	4	1	0	78.3	47.8	*P* = 1.000
							[56.3–92.5]	[26.8–69.4]	
2	15	7	4	1	1	2	73.3	46.7	
							[44.9–92.2]	[21.3–73.4]	
ECOG performance status									
0	14	9	4	1	0	0	92.9	64.3	*P* = 0.115
							[66.1–99.8]	[35.1–87.2]	
1	24	9	7	1	2	2	66.7	37.5	
							[44.7–84.4]	[18.8–59.4]	
Ann Arbor stage									
I–III	19	9	6	3	1	0	78.9	47.4	*P* = 1.000
							[54.4–93.9]	[24.4–71.1]	
IV	19	9	5	2	1	2	73.7	47.4	
							[48.8–90.9]	[24.4–71.1]	
Relapsed after HDC/ASCT									
	6	3	2	1	0	0	83.3 [35.9–99.6]	50.0 [11.8–88.2]	
Time-to-enrollment after the initiation of first-line therapy^b^									
< 12 months	7	1	2	3	1	0	42.9	14.3	*P* = 0.017
							[9.9–81.6]	[0.4–57.9]	
≥ 12 months	16	10	5	1	0	0	93.8	62.5	
							[69.8–99.8]	[35.4–84.8]	
Tumor size									
< 5 cm	28	14	9	2	2	1	82.1	50.0	*P* = 0.205
							[63.1–93.9]	[30.6–69.4]	
≥ 5 cm	10	4	2	3	0	1	60.0	40.0	
							[26.2–87.8]	[12.2–73.8]	
Cell of origin—gene expression profiling									
GCB	6	5	1	0	0	0	100	83.3	P=0.082
							[54.1-100.0]	[35.9-99.6]	
ABC	9	3	2	2	0	2	55.6	33.3	
							[21.2-86.3]	[7.5-70.0]	
Unclassified	5	2	3	0	0	0	100	40.0	
							[47.8-100.0]	[5.3-85.3]	
Cell of origin—Hans algorithm									
GCB	12	8	2	0	1	1	83.3	66.7	*P* = 1.000
							[51.6–97.9]	[34.9–90.1]	
Non-GCB	23	9	9	4	0	1	78.3	39.1	
							[56.3–92.5]	[19.7–61.5]	
Serum LDH, IU/L									
< 240	25	15	7	2	0	1	88.0	60.0	*P* = 0.040
							[68.8–97.5]	[38.7–78.9]	
≥ 240	13	3	4	3	2	1	53.8	23.1	
							[25.1–80.8]	[5.0–53.8]	
All patients	38	18	11	5	2	2	76.3 [59.8–88.6]	47.4 [31.0–64.2]	
Extranodal involvement									
< 2 lesions	34	16	9	5	2	2	73.5	47.1	*P* = 0.554
							[55.6–87.1]	[29.8–64.9]	
≥ 2 lesions	4	2	2	0	0	0	100.0	50.0	
							[39.8–100.0]	[6.8–93.2]	
IPI risk category—low, low-intermediate: score < 3; high-intermediate, high: score ≥ 3)									
< 3	29	17	8	3	0	1	86.2	58.6	*P* = 0.020
							[68.3–96.1]	[38.9–76.5]	
≥ 3	9	1	3	2	2	1	44.4	11.1	
							[13.7–78.8]	[0.3–48.2]	

^a^Calculated for the overall response rate according to Fisher’s exact test

^b^Includes eligible patients who received 1 regimen; other patients who received 2 regimens or ASCT were not included

*n* number of patients, *CR* complete response, *PR* partial response, *SD* stable disease, *PD* progressive disease, *NE* not evaluable, *ECOG* Eastern Cooperative Oncology Group, *ORR* overall response rate, *CI* confidence interval, *HDC/ASCT* high-dose chemotherapy/autologous stem cell transplantation, *GCB* germinal center B-cell-like, *ABC* activated B-cell-like, *LDH* lactate dehydrogenase, *IPI* international prognostic index

### Safety

Grade 3–5 treatment-emergent adverse events occurred in 100% of patients, and Table 3 summarizes TEAEs with an incidence of ≥ 10%. Regarding 389 episodes of hematologic TEAEs in 38 patients (100.0%), grade 3 and 4 hematologic TEAEs occurred in 7.9% and 92.1% of patients, respectively. Grade 4 hematologic TEAEs included lymphocyte count decreased (81.6%), CD4 lymphocytes decreased (52.6%), and neutrophil count decreased (26.3%). Concerning 394 episodes of nonhematologic TEAEs in 37 patients (97.4%), the relevant causality was determined with respect to 299 episodes in 34 patients (89.5%). Most (89.5%) of patients received at least 1 dose of G-CSF. Primary prophylactic use of G-CSF, which had been defined as its administration within 7 days from the beginning of each cycle, occurred in 4/38 (10.5%) of patients in cycle 1 and in 16/38 (42.1%) of patients in total (Supplementary Table S2). In most of the cases, G-CSF has been used for the secondary prophylactic use purpose in this setting. Patients—who received prophylactic G-CSF at least once and who did not—respectively developed the following TEAEs: grade 3–5 blood and lymphatic system disorders (MedDRA), 3 cases (18.8%) and 7 cases (31.8%); and grade 3–5 infections and infestations (MedDRA), 1 case (6.3%) and 6 cases (27.3%). A total of 15 cases (39.5%) of grade ≥ 3 nonhematologic TEAEs occurred, including 2 cases each (5.3%) of pharyngitis, pneumonia, appetite decreased, and hypertension. The total number of patients who developed opportunistic infections was 5 (13.2%—CMV 2, CMV enterocholitis 1, CMV viremia 1, and oral candidiasis 1). One case (2.6%) of grade 5 nonhematologic TEAE was CMV enterocolitis that developed during follow-up—day 176 from the last dosing of B.

**Table 3:** Summary of TEAEs (incidence: ≥ 10%) in the safety analysis set

Total number of TEAEs			783
Patients who had at least one TEAE (any causality), *n* (%)			38 (100.0)
TEAEs—grade ≥ 3, *n* (%)			38 (100.0)
Serious TEAEs, *n* (%)			13 (34.2)
TEAEs that led to treatment discontinuation, *n* (%)			17 (44.7)
Common TEAEs	All grades	Grade 3	Grade 4
Hematologic, *n* (%)	38 (100.0)		
Lymphocyte count decreased	34 (89.5)	3 (7.9)	31 (81.6)
Neutrophil count decreased	31 (81.6)	18 (47.4)	10 (26.3)
White blood cell count decreased	31 (81.6)	22 (57.9)	3 (7.9)
CD4 lymphocytes decreased	25 (65.8)	5 (13.2)	20 (52.6)
Platelet count decreased	25 (65.8)	3 (7.9)	5 (13.2)
Anemia	8 (21.1)	3 (7.9)	0 (0.0)
Febrile neutropenia	4 (10.5)	4 (10.5)	0 (0.0)
Hemoglobin decreased	4 (10.5)	0 (0.0)	0 (0.0)
Nonhematologic, *n* (%)	37 (97.4)		
Infusion-related reaction	13 (34.2)	0 (0.0)	0 (0.0)
Pyrexia	12 (31.6)	1 (2.6)	0 (0.0)
IgM decreased	12 (31.6)	0 (0.0)	0 (0.0)
IgG decreased	10 (26.3)	0 (0.0)	0 (0.0)
Constipation	10 (26.3)	0 (0.0)	0 (0.0)
Nausea	10 (26.3)	0 (0.0)	0 (0.0)
Decreased appetite	9 (23.7)	2 (5.3)	0 (0.0)
AST increased	9 (23.7)	0 (0.0)	0 (0.0)
Malaise	9 (23.7)	0 (0.0)	0 (0.0)
ALT increased	7 (18.4)	0 (0.0)	0 (0.0)
Hepatic function abnormal	6 (15.8)	0 (0.0)	1 (2.6)
γ-GTP increased	6 (15.8)	1 (2.6)	0 (0.0)
Rash	6 (15.8)	1 (2.6)	0 (0.0)
Vomiting	6 (15.8)	0 (0.0)	0 (0.0)
IgA decreased	6 (15.8)	0 (0.0)	0 (0.0)
Hyperkalemia	6 (15.8)	0 (0.0)	0 (0.0)
Renal impairment	5 (13.2)	0 (0.0)	1 (2.6)
CRP increased	5 (13.2)	0 (0.0)	0 (0.0)
Weight decreased	5 (13.2)	0 (0.0)	0 (0.0)
Vasculitis	5 (13.2)	0 (0.0)	0 (0.0)
Hypokalemia	4 (10.5)	0 (0.0)	1 (2.6)
Stomatitis	4 (10.5)	1 (2.6)	0 (0.0)
Blood creatinine increased	4 (10.5)	0 (0.0)	0 (0.0)
LDH increased	4 (10.5)	0 (0.0)	0 (0.0)
Taste disorder	4 (10.5)	0 (0.0)	0 (0.0)
Pruritus	4 (10.5)	0 (0.0)	0 (0.0)

Terms: expressed according to Medical Dictionary for Regulatory Activities Japanese version 22.1

Grades: assessed according to CTCAE version 4.0-JCOG

*TEAEs* treatment-emergent adverse events, *n* number of patients, *CTCAE* common terminology criteria for adverse events, *JCOG* Japan Clinical Oncology Group, *Ig* immunoglobulin, *ALT* alanine aminotransferase, *AST* aspartate aminotransferase, *γ-GTP* gamma-glutamyl transpeptidase, *CRP* C-reactive protein, *LDH* lactate dehydrogenase

## Discussion

The present single-arm, phase 3 study basically confirmed the efficacy results and indicated the safety profile of BR similar to that described in our phase 2 study of BR [6] which had been conducted according to the same dosing schedule as the present study in 59 patients with rrDLBCL—whose median age was 67 years (range, 36–75) and who had undergone 1 to 3 prior chemotherapy regimens; the median of delivered cycles was 4 (range, 1–6). Briefly, our phase 2 study reported an ORR of 62.7% (95% CI, 49.1–75.0%), a CR rate of 37.3% (95% CI, 25.0–50.9%), a median PFS of 6.7 months (95% CI, 3.6–13.7 months), and the most common grade 3–4 hematologic TEAEs of lymphopenia, neutropenia, leukopenia, CD4 lymphopenia, and thrombocytopenia. In designing the present study, we set an expected ORR of 62% to confirm the efficacy observed in the previous phase 2 study (ORR of 62.7%), and the ORR of 76.3% (95% CI: 59.8–88.6; Table 2) in the current study met the primary endpoint. The difference in the eligibility criteria (e.g., prior lines of therapy 1–2 vs 1–3) could favor the current study. Other criteria such as the refractoriness to the prior regimen was identical to those of the phase 2 study. Since the primary purpose of the study was to test the result of the past phase 2 study, the primary endpoint was set in this study identically with that of the past study. Additionally, the long-term efficacy of this BR regimen was demonstrated by the long-term follow-up survey of the present study that indicated a median OS of as long as 29.2 months. With regard to the argument around primary endpoint for rrDLBCL, survival should be appreciated as the true endpoint in general. Nevertheless, long-term survey of the patients requires a long study term. In addition, the impact of the subsequent treatment may matter when evaluating the efficacy. Better response is likely to lead to better prognosis observed in this study which may suggest the value of response as the primary endpoint of the clinical trial for rrDLBCL. The safety profile of the present study was in line with that of our phase 2 study [6]. Of note were the facts that this salvage chemotherapy regimen was effective for elderly, ASCT-ineligible patients with rrDLBCL including a history of HDC/ASCT although the sample size was very small and that many patients were responsive to this BR regimen in a few cycles. In 2016, an international consensus panel of B experts published a set of updated recommendations on the safe and effective use of B in patients with hematologic disorders including rrDLBCL [12] based on clinical evidence obtained from our phase 2 study and the phase 2 study of BR in patients with rrDLBCL conducted by Vacirca et al. [13]. The panel recommended the cautious administration of B to patients with rrDLBCL, with a dose of 90–120 mg/m^2^ given on 2 consecutive days combined with R every 3 weeks for 4–6 cycles and a dose de-escalation of 120–90–70 mg/m^2^ on days 1 and 2 in cases of toxicity. Eleven patients (28.9%) completed 6 cycles in the present study, supporting these recommendations. The present phase 3 study, which met its objective of confirming the results from our phase 2 study, reverified the occurrence of treatment delay and dose reduction when initiating the next cycle in a certain proportion of patients as observed in the phase 2 study. In the clinical settings, B 90 mg/m^2^ may preferably be an alternative dose option to be taken based on patient condition as described in the panel’s recommendations. Despite most of patients with rrDLBCL received G-CSF and preventive measures for opportunistic infections were recommended for patients whose CD4 lymphocyte count was ≤ 200/mm^3^, the total number of patients who developed opportunistic infections was 14 (23.7%—CMV infections 6, herpes virus infections 4, herpes zoster virus infections 4) in the phase 2 study and was 5 (13.2%) in the present study. This might be due to a difference in dexamethasone use as prophylaxis for chemotherapy-induced nausea and vomiting. In fact, in the previous phase 2 study, dexamethasone 20 mg IV on days 1–3 and 10 mg orally on days 4–5 was used for prophylaxis for chemotherapy-induced nausea and vomiting, which might have been overdosed. In contrast, prophylaxis for emesis with steroids (e.g., dexamethasone) was not predefined in the present study.

Sehn et al. conducted a randomized phase 2 study of a novel anticancer agent CD79b-directed antibody–drug conjugate, polatuzumab vedotin, combined with BR (pola-BR) in patients with ASCT-ineligible rrDLBCL [8]. They reported the following efficacy outcomes for the cohort of polatuzumab vedotin 1.8 mg/kg IV plus B 90 mg/m^2^ IV—a dose lower than B 120 mg/m^2^ IV used in the present study—and R 375 mg/m^2^ IV (the pola-BR arm) against the cohort of polatuzumab 1.8 mg/kg IV and B 90 mg/m^2^ IV (the BR arm): the CR rate, 40.0% vs. 17.5%; median PFS, 9.5 vs. 3.7 months; and median OS, 12.4 vs. 4.7 months. Patients in the pola-BR arm had the higher incidences of grade 3–4 neutropenia (46.2% vs. 33.3%), anemia (28.2 vs. 17.9%), and thrombocytopenia (41.0% vs. 23.1%), while the incidences of grade 3–4 infections were similar (23.1% vs. 20.5%) between the pola-BR and BR arms. The median numbers of completed cycles in the pola-BR arm and the BR arm were 5 and 3, respectively—primarily due to a higher rate of PD in the BR arm. B dose reduction, treatment delay, and treatment discontinuation due to PD in the BR arm were 10.3%, 38.5%, and 53.8% against 12.8%, 53.8%, and 15.4% in the pola-BR group, respectively.

BR-based, salvage chemoimmunotherapy regimens that are currently available for patients with rrDLBCL are the BR regimen using B 120 mg/m^2^ as the initial dose and the add-on regimen of pola-BR using the dose of B 90 mg/m^2^. The present study enrolled patients with rrDLBCL who underwent up to 2 prior chemotherapy regimens. Therefore, a high response can be expected for the BR regimen using B 120 mg/m^2^ in most of patients with rrDLBCL who have undergone less lines of prior treatment. The pola-BR study and the present study are difficult to compare because of differences in patient background (e.g., number of prior chemotherapy regimens and refractoriness to the last treatment), and assessment procedures (e.g., timing for response assessment).

In the present study, we verified that the BR regimen using B 120 mg/m^2^ has promising efficacy and an acceptable safety profile for patients who are not refractory to any of prior treatments and who presented the first or second relapse. Primary prophylaxis for cytopenia, infections, and emesis is a reasonable management of patients with rrDLBCL who undergo the BR regimen.

The present study has several limitations. First, the present study was not a randomized study but a single-arm, small-sized study with little ethnic diversity, which was decided through consultation with the health authority of Japan. Second, the present study included only relapsed patients with 1–2 prior lines of therapy and patients with refractory disease and/or patients who were heavily pretreated were not eligible, and previously heavily treated patients were not eligible. Third, the difficulty in inter-study comparisons impedes the determination of superiority of BR to other regimens and of the optimal sequences to implement different treatments in combination.

In conclusion, the present study confirmed the results of our phase 2 study and demonstrated the favorable efficacy and an acceptable safety profile of the BR regimen using the initial dose of B 120 mg/m^2^ as a second- or third-line salvage chemotherapy regimen in HDC/ASCT-ineligible patients with rrDLBCL.

## Supplementary Information

Below is the link to the electronic supplementary material.

Supplementary file1 (PDF 544 kb)

## Data Availability

The datasets generated during and/or analyzed during the current study are not generally available due to the lack of consent for the disclosure of the datasets to the public, but may be available from the corresponding author on reasonable request in some cases.
