# Supplementary material for: Bendamustine plus rituximab in Japanese patients with relapsed or refractory diffuse large B-cell lymphoma
Source: Ann Hematol. 2022 Mar 4;101(5):979–89. doi: 10.1007/s00277-022-04801-2 (PMC8993776; doi:10.1007/s00277-022-04801-2)
Supplement: Supplementary file 1 — Supplementary file1 (PDF 544 kb) [file 277_2022_4801_MOESM1_ESM.pdf]

Supplementary Figure S1

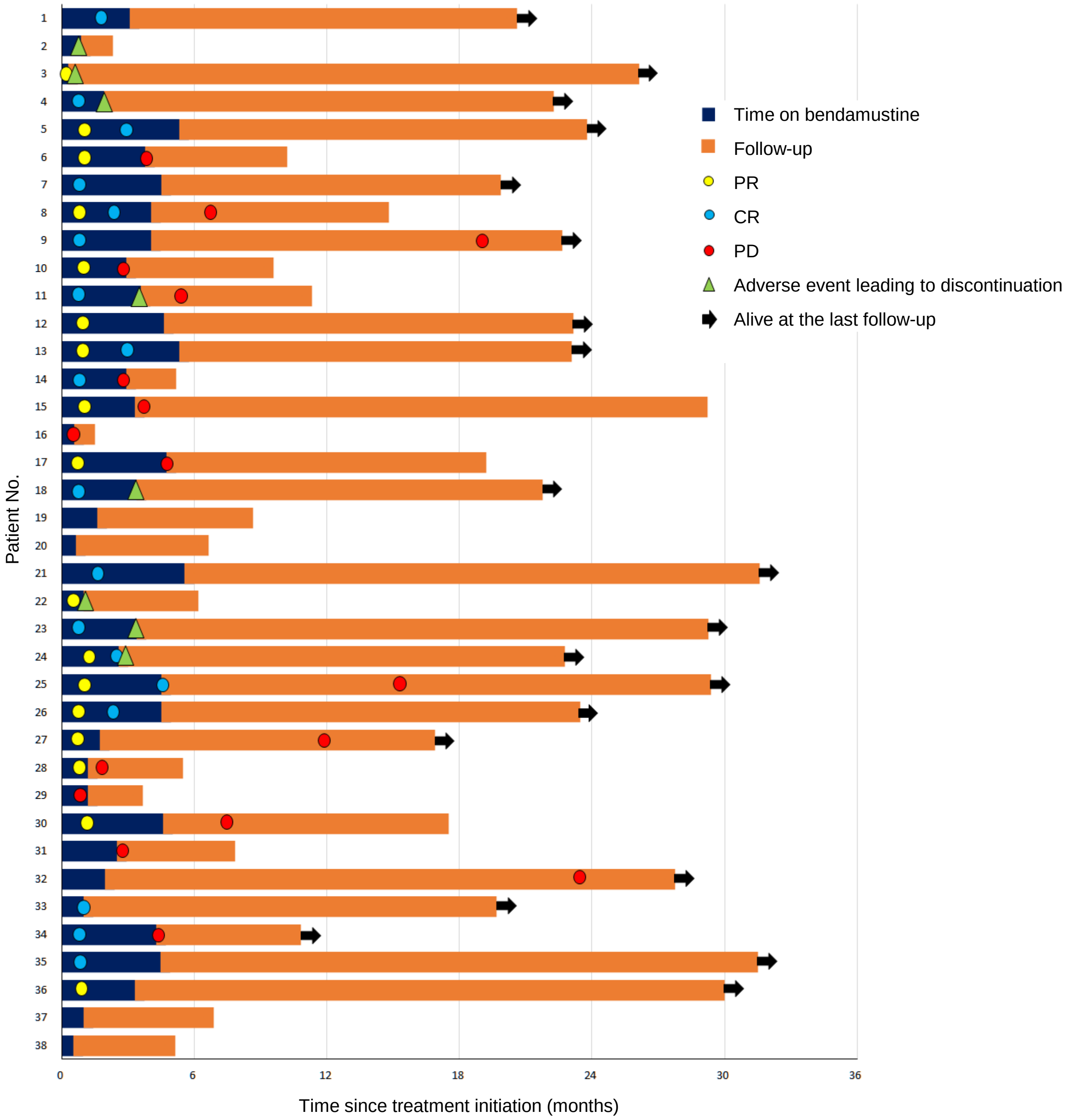

Supplementary Table S1. Exposure to the BR regimen.

	Delivered cycles: median 4 [range, 1-6]					
	Cycle 1	Cycle 2	Cycle 3	Cycle 4	Cycle 5	Cycle 6
Patients who received BR, n (%)	38 (100)	29 (76.3)	24 (63.2)	20 (52.6)	15 (39.5)	11 (28.9)
Treatment delay when proceeding to the next cycle, n[a]	7	14	1	7	4	–
Reduction in B dose when proceeding to the next cycle, n[a]	4[b]	8[c]	0	6[d]	3[e]	–
RDI[f]	Delivered cycles					
	Cycle 1	Cycle 2	Cycle 3	Cycle 4	Cycle 5	Cycle 6
n	38	29	24	20	15	11
Mean, %	89.4	80.1	82.1	78.1	69.1	72.5
Median, %	97.9	77.5	84.2	74.1	73.5	72.9
Dose level	Delivered cycles, n					
	Cycle 1	Cycle 2	Cycle 3	Cycle 4	Cycle 5	Cycle 6
60 mg/m^2	0	0	1	1	4	3
90 mg/m^2	0	4	9	7	4	5
120 mg/m^2	38	25	14	12	7	3

–: not applicable

[a]: Represents the number of patients in the corresponding cycle of the each raw.

[b]: from 120 mg/m^2 to 90 mg/m^2 for all 4 patients

[c]: from 120 mg/m^2 to 90 mg/m^2 for 7 patients and from 90 mg/m^2 to 60 mg/m^2 for 1 patient

[d]: from 120 mg/m^2 to 90 mg/m^2 for 3 patients and from 90 mg/m^2 to 60 mg/m^2 for 3 patients

[e]: from 120 mg/m^2 to 90 mg/m^2 for all 3 patients

[f]: defined as DDI/SDI × 100%

DDI: (delivered total dose, in mg/m^2)/(actual time to complete chemotherapy with imputation for missed cycles, in days)

SDI: (standard total dose, in mg/m^2)/(standard time to complete chemotherapy, in days)

BR, bendamustine-rituximab; RDI, relative dose intensity; DDI, delivered dose intensity; SDI, standard dose intensity

Supplementary Table S2. Use of G-CSF by cycle in patients of the safety population

Short-acting G-CSF and/or Peg G-CSF		Short-acting G-CSF			
Cycle	Number of patients who received G-CSF/number of remaining patients	Cycle	Number of patients who received G-CSF/number of remaining patients	Number of days to administration onset	Number of administration*
				Median Minimum–maximum	Median Minimum–maximum
1	13/38	1	11/38	15.0 (4–30)	5.0 (1–14)
2	22/29	2	17/29	11.0 (5–30)	3.0 (1–10)
3	16/24	3	6/24	9.5 (7–17)	4.5 (1–9)
4	15/20	4	6/20	15.0 (8–23)	7.5 (1–13)
5	13/15	5	5/15	11.0 (8–26)	4.0 (2–10)
6	9/11	6	2/11	9.0 (8–10)	1.0 (1-1)
Total	34/38 (89.5%)	Total	30/38 (78.9%)		

Short-acting G-CSF and/or Peg G-CSF		Peg G-CSF			
Cycle	Number of patients who received prophylactic G-CSF/number of remaining patients	Cycle	Number of patients who received peg G-CSF/ number of remaining patients	Number of days to administration onset	Number of administration*
				Median Minimum–maximum	Median Minimum–maximum
1	4/38	1	2/38	6.0 (5–7)	1 (1–1)
2	9/29	2	6/29	4.5 (4–7)	1 (1–1)
3	10/24	3	10/24	5.0 (4–8)	1 (1–2)
4	9/20	4	9/20	5.0 (4–7)	1 (1–1)
5	8/15	5	8/15	4.5 (4–7)	1 (1–2)
6	6/11	6	7/11	5.0 (4–14)	1 (1–1)
Total	16/38 (42.1%)	Total	12/38 (31.6%)		

*Total of administration in each cycle

G-CSF, granulocyte colony-stimulating factor; Peg G-CSF, pegylated granulocyte colony-stimulating factor
